# Early Metabolic Benefits of Switching Hydrocortisone to Modified Release Hydrocortisone in Adult Adrenal Insufficiency

**DOI:** 10.3389/fendo.2021.641247

**Published:** 2021-03-11

**Authors:** Christopher A. M. Bannon, Daniel Border, Petra Hanson, John Hattersley, Martin O. Weickert, Ashley Grossman, Harpal S. Randeva, Thomas M. Barber

**Affiliations:** ^1^ Warwickshire Institute for the Study of Diabetes Endocrinology and Metabolism, University Hospitals Coventry and Warwickshire, Clifford Bridge Road, Coventry, United Kingdom; ^2^ Warwick Medical School, University of Warwick, Coventry, United Kingdom; ^3^ NIHR CRF Human Metabolism Research Unit, University Hospitals Coventry and Warwickshire NHS Trust, Coventry, United Kingdom; ^4^ Faculty of Health & Life Sciences, Centre of Applied Biological & Exercise Sciences, Coventry University, Coventry, United Kingdom; ^5^ Neuroendocrine Tumour Unit, Royal Free Hospital, London, United Kingdom; ^6^ Oxford Centre for Diabetes, Endocrinology and Metabolism, University of Oxford, Oxford, United Kingdom

**Keywords:** adrenal insufficiency, modified-release hydrocortisone, metabolism, hydrocortisone, fat mass

## Abstract

**Purpose:**

To compare metabolic effects of modified release hydrocortisone (MR-HC) with standard hydrocortisone (HC) therapies in adults with Adrenal Insufficiency (AI).

**Methods:**

Adult patients (n = 12) with AI, established on HC therapy, were recruited from Endocrinology clinics at University Hospitals Coventry and Warwickshire (UHCW), UK. Baseline (HC) metabolic assessments included fasting serum HbA1C, lipid and thyroid profiles, accurate measures of body composition (BodPod), and 24-h continuous measures of energy expenditure including Sleeping Metabolic Rate (SMR) using indirect calorimetry within the Human Metabolism Research Unit, UHCW. All participants then switched HC to MR-HC with repeat (MR-HC) metabolic assessments at 3 months. Paired-sample t-tests were used for data comparisons between HC and MR-HC assessments: P-value <0.05 was considered significant.

**Results:**

Following exclusion of 2 participants, analyses were based on 10 participants. Compared with baseline HC data, following 3 months of MR-HC therapy mean fat mass reduced significantly by −3.2 kg (95% CI: −6.0 to −0.4). Mean (SD) baseline HC fat mass vs repeat MR-HC fat mass: 31.9 kg (15.2) vs 28.7 kg (12.8) respectively, P = 0.03. Mean SMR increased significantly by +77 kcal/24 h (95% CI: 10–146). Mean (SD) baseline HC SMR vs repeat MR-HC SMR: 1,517 kcal/24 h (301) vs 1,594 kcal/24 h (344) respectively, P = 0.03. Mean body fat percentage reduced significantly by −3.4% (95% CI: −6.5 to −0.2). Other measures of body composition, energy expenditure, and biochemical analytes were equivalent between HC and MR-HC assessments.

**Conclusions:**

In adults with AI, switching from standard HC to MR-HC associates with early metabolic benefits of reduced fat mass and increased SMR.

## Introduction

Adrenal insufficiency (AI) is a chronic and often lifelong disease that can affect the hypothalamo-pituitary adrenal (HPA) axis at any level (1). On a population level, AI is relatively uncommon, with a prevalence of 93 to 144 per million (primary AI) and 150 to 280 per million (secondary AI, including those patients with suppression of the HPA axis from chronic use of exogenous corticosteroids in supra-physiological doses) (2). The effective management of patients with AI, regardless of its etiology, includes glucocorticoid replacement therapies (GRTs), as well as mineralocorticoid replacement with primary AI. Any interruption or insufficiency of GRT (such as during acute infections, trauma or surgery) can manifest in acute deterioration of clinical status (adrenal crisis), and threaten life (3).

Hydrocortisone (HC) is the most common form of GRT in the UK and worldwide (4). Unfortunately, HC has a relatively short half-life (around 120 min for total cortisol and 90 min for free cortisol) (5, 6). Accordingly, most patients who take HC require three doses per day, with the last dose often taken late afternoon. The aim of any GRT regimen is to replicate the normal circadian rhythm of serum cortisol which has its zenith early in the morning just before waking (following a surge in the 1 to 2 h before waking), with levels gradually dropping during the day and at night (7). Unfortunately, the use of standard HC thrice daily results in three ‘mini-peaks’ of steroid following each dose. Furthermore, there may be a period of 12 to 16 h (late evening and during the night) where no HC is taken such that steroid levels can drop precipitously, sometimes to dangerously low levels in patients who have completely insufficient endogenous glucocorticoid production. Finally, the short half-life of HC can increase the vulnerability of patients with AI to adrenal crisis, with delayed or missed doses. Compared to controls with a normal HPA axis, patients with AI on HC therapy have a higher standardized mortality rate (8, 9), and an increased risk of weight gain and dysglycemia (10). The pathogenesis of the increased mortality and morbidity of patients with AI on HC therapy is incompletely understood, and likely contributed to by multiple factors. One such factor may stem from the mismatch between a typical daily steroid profile of AI patients on multiple daily doses of HC therapy, and a normal circadian rhythm of cortisol from an intact HPA axis.

More recently, an alternate form of HC therapy has become available for the management of AI. Modified-release Hydrocortisone (MR-HC) differs from HC in that its long half-life enables once-daily administration, thereby reducing the vulnerability from steroid insufficiency from delayed or missed (multiple) doses (11, 12). MR-HC has a ‘dual-release’ mechanism whereby HC within the outer layer of the tablet is released immediately following ingestion. This is then followed by a slow and sustained release of HC from within the core of the tablet (13). Compared with thrice daily HC, MR-HC provides a more accurate reflection of the normal diurnal rhythm of cortisol (12), but fails to match the physiological nocturnal rise of serum cortisol (14). Furthermore, in a recent meta-analysis of the current literature comparing HC with MR-HC in AI, our own group showed that MR-HC associates with a more favorable metabolic outcome, including reduced Body Mass Index (BMI) and waist circumference, and improved glycemic control (15–18). The underlying mechanism(s) that mediate the metabolic improvements from MR-HC remain contentious and incompletely understood. However, one intriguing hypothesis relates to the possible metabolic effects of GRTs on clock gene expression. Accordingly, Venneri and colleagues showed that in patients with AI, thrice daily HC treatment associated with disruption of the expression of certain circadian clock genes, with restored clock gene expression within 3 months of switching from HC to MR-HC therapy (19).

The emergence of MR-HC has provided an alternate once-daily GRT treatment option in patients with AI, with superior replication of the physiological profile of serum cortisol compared with the more traditional option of multiple daily HC administration. It is perhaps surprising, therefore, that the current literature lacks any detailed metabolic comparisons between these two treatment modalities in AI, other than the fairly blunt anthropometric criteria of BMI and waist circumference (15). Our objective was to explore the more detailed metabolic effects of MR-HC in patients with AI following a switch from multiple daily HC therapy, in the most highly-phenotyped study reported on to date.

## Methods

### Recruitment

We recruited adult patients (aged ≥18 years) with a confirmed diagnosis of AI (n = 12) on an established regimen of HC, administered twice or thrice daily. Recruitment (between August 2018 and April 2019) was from general endocrinology clinics at the Warwickshire Institute for the Study of Diabetes, Endocrinology and Metabolism (WISDEM) Centre, University Hospitals Coventry and Warwickshire (UHCW), UK. Only those patients who had been considered for switch to MR-HC for clinical purposes were recruited, the reasons including need for improved compliance with reduced pill burden, improved corticosteroid coverage during the late evening and night and patient preference. This was a pilot, observational study, and therefore lacked any randomization or placebo arm. Exclusion criteria included therapies (such as beta-adrenoceptor blockers) that could interfere with body weight and energy expenditure. All participants provided fully informed written consent prior to enrolment into the study. All clinical investigations were conducted in accordance with the guidelines in the declaration of Helsinki. The study had specific approval from the West Midlands Black Country Research Ethics Committee, UK.

### Participant Pathway

**Figure 1:**
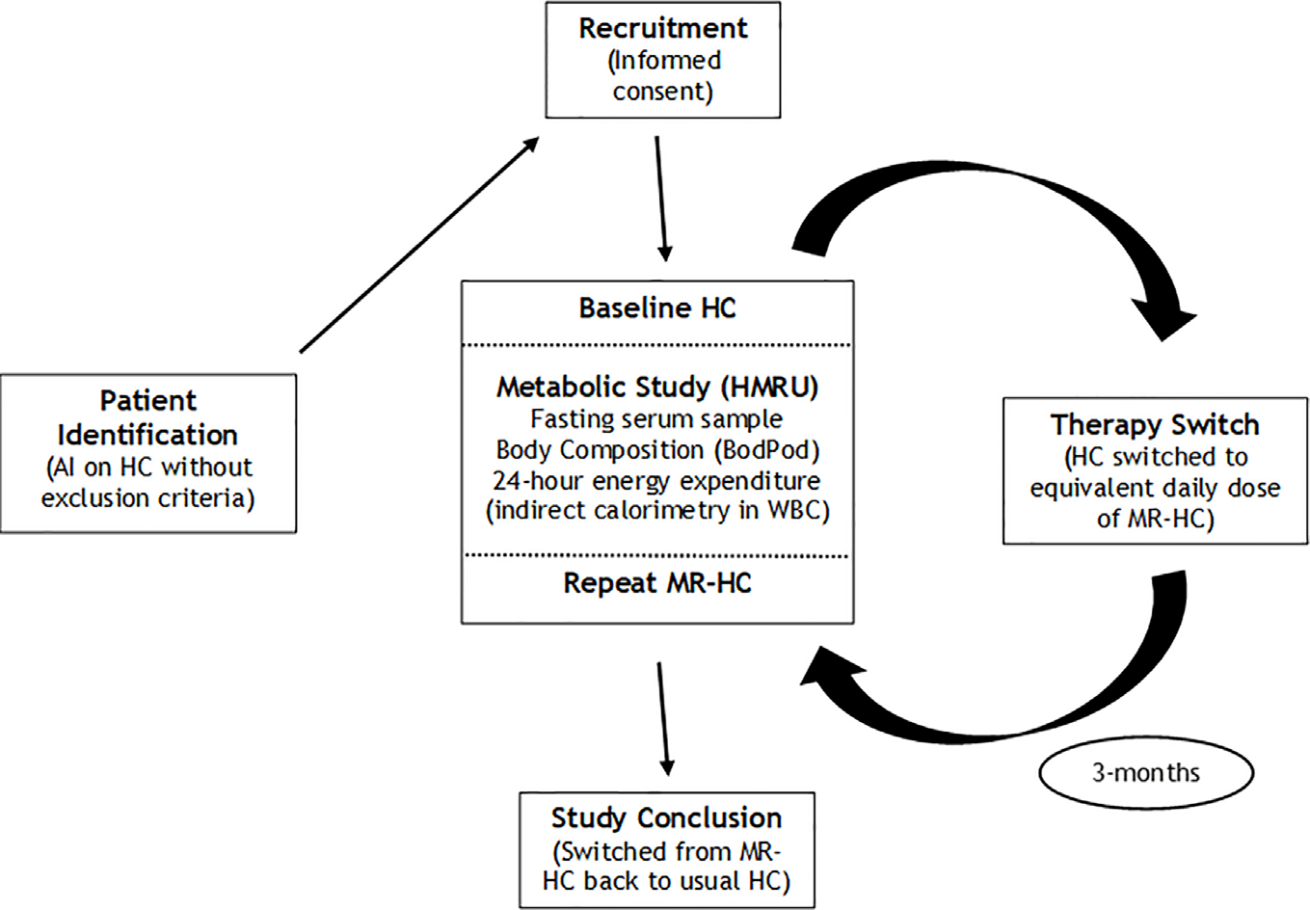
Summary of research pathway AI, adrenal Insufficiency; HC, hydrocortisone; HMRU, Human Metabolism Research Unit; MR-HC, modified release hydrocortisone; WBC, whole body calorimeter.

Following enrolment, each participant was invited to attend for a baseline (‘HC’) metabolic assessment (whilst taking their usual HC therapy), to include a fasting blood test, accurate measures of body composition and 24-h continuous energy expenditure through indirect calorimetry within a whole-body calorimeter (WBC) at the Human Metabolism Research Unit (HMRU) at UHCW. Details of the WBC have been described previously (20). Following baseline metabolic assessment, each participant was switched from their HC to MR-HC. Patients were converted from HC to MR-HC, with the dose of MR-HC being equivalent to the total daily dose of HC (rounding up to a multiple of 5 mg, where necessary). Following 3 months of treatment with MR-HC, each participant was invited to have a repeat (‘MR-HC’) metabolic assessment on HMRU, to include the same measures as indicated above. The repeat metabolic assessment marked the conclusion of the study, and all participants were then switched back to their usual HC therapies, and had usual clinical follow-up thereafter within the Endocrine clinics at WISDEM center at UHCW, UK. An outline summary of the research pathway is shown in **Figure 1**.

### Assessments of Body Composition and Energy Expenditure

As indicated, each participant had two separate metabolic assessments on HMRU: i) Baseline HC, and; ii) Repeat MR-HC. For each participant, the two metabolic studies were equivalent. On their assigned metabolic study day, participants were required to arrive at HMRU for 8:00 am in a fasting state (no food or drink other than water for 8 h). Each participant was required to take their HC (for baseline assessment) or MR-HC (for repeat assessment) at the usual times throughout each of the metabolic study days (including any early morning doses prior to travelling to HMRU). All participants were required to maintain a stable isocaloric diet for 3-days prior to each metabolic study day. Compliance with pre-metabolic study isocaloric diet was verified using a self-completed three-day dietary questionnaire (provided to each participant at enrolment). Furthermore, all participants were required to avoid strenuous physical activity and caffeine ingestion for a period of 24 h prior to each metabolic study.

Each metabolic study commenced with acquisition of fasting blood samples, followed by anthropometric assessment of body composition (including fat and lean mass) using a BodPod (Cosmed Inc., USA), a technique described previously and based on air displacement plethysmography (21). Following a fasting blood test and anthropometric assessment, each participant was then invited to enter the WBC at 9:00 am for a 24-h continuous assessment of energy expenditure. Whilst inside the WBC, participants were requested to avoid physical activity. Temperature (thermo-neutrality at 24°C) and relative humidity (57%) were kept constant within the WBC throughout all metabolic studies, to limit any potential for confounding effects. Whilst inside the WBC, standard meals were provided at pre-determined times that were kept constant throughout all metabolic studies: Breakfast at 09:30hrs; Lunch at 12:15hrs; Dinner at 17:00hrs, and; Evening snack at 20:00hrs. Macronutrient content of each meal was kept constant, and based on a standard diet of 35% fat, 15% protein, and 50% carbohydrate. Each participant was requested to sleep between 22:00hrs and 07:00hrs the next day. Following 24 h within the WBC, each participant was requested to exit the WBC at 09.00, marking the conclusion of the metabolic study. Following provision of breakfast, participants were able to go home.

Accurate minute-by-minute measurements of CO_2_ and O_2_ from air entering and leaving the WBC enabled continuous 24-h assessment of energy expenditure through application of Weir’s formula, described previously (20). Total Metabolic Rate (TMR) was a cumulative assessment derived from energy expenditure over the entire 24 h within the WBC. Sleeping metabolic rate (SMR) was defined as mean average energy expenditure (kcal per minute) during sleep (between 24.00hrs and 06.00hrs), multiplied by 1,440 to enable direct comparison with TMR over a 24-h period.

### Assessment of Biochemistry

Fasting blood samples were obtained from each participant at baseline HC and repeat MR-HC metabolic assessments. Following immediate spinning of each blood sample in a centrifuge, serum was extracted and analyzed for HbA1c, lipid profile (total cholesterol, low-density lipoprotein [LDL] cholesterol, high-density lipoprotein [HDL] cholesterol and triglycerides) and thyroid function parameters (Thyroid Stimulating Hormone [TSH], free T4 and free T3). HbA1C was assayed using a TOSOH G8 analyzer (high performance liquid chromatography method), with a lower limit of detection of 3 mmol/mol. Lipid parameters were assayed using a Roche/Hitachi cobas c system (enzymatic, calorimetric method), with a lower limit of detection of 0.1 mmol/L (3.86 mg/dl) for total cholesterol, and 0.1 mmol/L (8.85 mg/dl) for triglyceride. Thyroid function parameters were assayed using an Elecsys and a cobas e immunoassay analyzer.

### Statistical Analyses

All statistical analyses were performed using Stata (version 15.0). Normality for each variable was tested using Shapiro-Wilk test, with a p-value of ≤0.05 deemed significant. For all normally-distributed variables, paired-sample t-tests were used to compare data for each participant from their baseline HC and repeat MR-HC metabolic assessments. Using each participant as their own comparator with the paired-sample t-test limits any confounding effects. For BMI (the only non-normally distributed variable), the Wilcoxon Sign Test was used for comparison baseline HC and repeat MR-HC metabolic assessments, with a p-value of <0.05 deemed significant. Data are reported as mean and standard deviation (SD) with 95% confidence intervals for normally distributed variables. As a non-normally distributed variable, BMI is reported as median and inter-quartile range (IQR). As this was an exploratory study, it was not possible to perform any *a priori* power calculations.

## Results

### Descriptive Data for the Cohort

We recruited 12 patients with AI from the WISDEM Centre, UHCW. Of these 12 recruited participants, we excluded one of them due to their admission to hospital with an adrenal crisis during the 3-month study period. We also excluded another participant who needed to take additional doses of HC (for clinical purposes) combined with their MR-HC during the 3-month study period. We therefore included 10 participants in our analyses. Of those participants included in the analyses, one required an increased dose of thyroxine during the study, although they remained biochemically euthyroid throughout. (Sub-group analyses based on data that excluded this individual participant made no appreciable difference to the analyses or conclusions on body composition and energy expenditure data from the whole cohort, presented below).

Of the 10 participants included in the analyses, mean age was 54.2 years (SD, 21.2 years), and there were four women and six men. Administration of HC therapy was either thrice daily (n = 8) or twice daily (n = 2). Mean total daily dose of HC at enrolment was 23 mg (SD 6.0). The types of AI included primary (n = 6) and secondary (n = 4). One participant had Type 2 Diabetes Mellitus (T2D), and one had Type 1 Diabetes Mellitus (T1D), while the remaining 8 participants had no prior or current history of Diabetes Mellitus (DM) and had confirmed euglycemia. For the 4 participants with secondary AI, other hormone replacements included thyroxine (dose range 100–150 mcg per day; n = 3), desmopressin (n = 1), testosterone (*Tostran* gel; n = 2), and oestrogen replacement therapies (Loestrin, 20μg oestrogen, n = 1). For all participants on hormone replacement therapies, these were adequately replaced at enrolment, and throughout the entire study (confirmed through relevant biochemical measurements at baseline [HC] and follow-up [MR-HC] assessments). Two of the participants with primary AI had concurrent hypothyroidism with thyroxine replacement (adequately replaced). None of the participants with primary AI had any other concurrent endocrine autoimmune comorbidities or hormone replacement therapies.

### Body Composition

Compared with baseline HC data, there was a significant reduction in fat mass (mean: −3.2 kg; 95% CI: −6.0 to −0.4) following 3 months of MR-HC therapy (mean [SD] baseline HC fat mass vs repeat MR-HC fat mass: 31.9 kg [15.2] vs 28.7 kg [12.8] respectively, P = 0.03). Compared with baseline HC data, there was also a significant reduction in body fat percentage (mean: −3.4%; 95% CI: −6.5 to −0.2) following 3 months of MR-HC therapy (mean [SD] baseline HC body fat percentage vs repeat MR-HC body fat percentage: 37.8% [13.3] vs 34.4% [10.6] respectively, P = 0.04). All other measures of body composition, including BMI, total body weight, and lean mass were equivalent between baseline HC and repeat MR-HC metabolic assessments (data shown in **Table 1**; Individual data for each of the participants [n = 10] included in the analyses, including descriptive data, are shown in **Table 2**).

**Table 1:** Body composition, energy expenditure and biochemistry at baseline (HC) and follow-up (MR-HC) metabolic assessments.

Phenotype	Baseline on HC replacement (n = 10)	Follow-up on MR-HC replacement (n = 10)	Test statistic
**Body Composition**			
**BMI** (kg/m^2^)	26.3 (3.7)^α^	25.6 (4.4)^α^	NS^β^
**Bodyweight** (kg)	83.9 (17.2)	83.2 (18.2)	NS
**Fat Mass** (kg)	31.9 (15.2)	28.7 (12.8)	0.03*
**Body Fat** (%)	37.8 (13.3)	34.4 (10.6)	0.04*
**Lean Mass** (kg)	52.1 (15.8)	54.6 (15.3)	NS
**Energy Expenditure**			
**SMR** (kcal over 24 h)	1,517 (301)	1,594 (344)	0.03*
**SMR/LM** (kcal/kg lean mass over 24 h)	30.1 (5.1)	29.7 (3.1)	NS
**TMR** (kcal over 24 h)	1,980 (390)	2,006 (399)	NS
**TMR/LM** (kcal/kg lean mass over 24 h)	39.5 (7.7)	37.5 (4.8)	NS
**Biochemistry** (fasting serum samples)			
**HbA1C** (mmol/mol)	43.1 (14.6)	41.5 (13.0)	NS
**Total cholesterol** (mmol/L)	4.7 (0.8)	4.9 (0.7)	NS
**HDL-cholesterol** (mmol/L)	1.5 (0.3)	1.5 (0.3)	NS
**LDL-cholesterol** (mmol/L)	2.7 (0.7)	2.7 (0.5)	NS
**Triglycerides** (mmol/L)	1.3 (0.6)	1.5 (0.5)	NS
**Thyroid stimulating hormone** (mU/L)	1.7 (1.6)	1.5 (2.0)	NS
**Free T4** (pmol/L)	18.0 (3.0)	19.4 (3.2)	NS

Data presented as mean and (standard deviation) unless otherwise indicated. ^α^=median and Inter-Quartile Range; Test statistic indicates paired-sample t-test comparison between baseline and follow-up data unless otherwise indicated. ^β^=Wilcoxon sign test used as paired test.

BMI, Body Mass Index; HC, Hydrocortisone; HDL, High Density Lipoprotein; LDL, Low Density Lipoprotein; LM, Lean Mass; MR-HC, Modified Release Hydrocortisone; NS, Non-significant; SMR, Sleeping Metabolic Rate; TMR, total metabolic rate; *statistical significance. Bold p-values indicate statistical significance.

**Table 2:** Individual data for each of the participants (n = 10) included in the analyses.

ParticipantNumber	Age at recruitment(years)	Sex	Type of AI	Total daily dose of HC (mg)	Frequency HC admin(per day)	Daily dose of MR-HC (mg)	Percentage change FM with MR-HC^α^	Percentage change LM with MR-HC^α^	Percentage change SMR with MR-HC^α^	Percentage change TMR with MR-HC^α^	Comorbidities
1	47	M	P	30	2	30	+1.7	−2.6	+5.5	+7.7	T2D; Hypertension Stroke; CKD Dyslipidaemia
2	66	F	P	35	3	35	−17.7	+9.4	+11.0	+10.6	Atrial Fibrillation
3	66	M	P	15	2	20	−12.7	+27.3	+11.8	+3.3	Hypothyroidism Obesity; Asthma
4	37	F	P	22.5	3	25	−22.2	+12.4	+2.4	+0.7	Hypothyroidism
5	26	M	P	20	3	20	−1.9	+2.5	+5.8	+3.3	**-**
6	54	M	P	25	3	25	−1.9	−1.6	+3.7	−4.8	Hypertension Depression
7	21	F	S	17.5	3	20	−22.2	+12.4	−4.0	−2.2	**-**
8	87	M	S	20	3	20	+2.2	−2.8	+18.5	+6.1	**-**
9	70	M	S	25	3	25	−9.8	+0.9	0	−4.5	Hypertension
10	68	F	S	20	3	20	+2.2	0	−4.5	−5.3	T1D Dyslipidaemia

^α^= Data presented as a percentage of baseline values from HC metabolic study.

AI, adrenal insufficiency; BMI, body mass index; CKD, chronic kidney disease; F, female; FM, fat mass; HC, hydrocortisone; LM, lean mass; M, male; MR-HC, modified release hydrocortisone; P, primary; S, secondary; SMR, sleeping metabolic rate; T1D, type 1 diabetes mellitus; T2D, type 2 diabetes mellitus; TMR, total metabolic rate.

### Energy Expenditure

Compared with baseline HC data, there was a significant increase in SMR (mean: +77 kcal/24 h; 95% CI: 10–146) following 3 months of MR-HC therapy (mean [SD] baseline HC SMR vs repeat MR-HC SMR: 1,517 kcal/24 h [301] vs 1,594 kcal/24 h [344] respectively, P = 0.03). When lean mass (LM) was factored into SMR comparisons (using LM as a divisor), SMR/LM for baseline HC and repeat MR-HC metabolic assessments became equivalent. TMR and TMR/LM were equivalent between baseline HC and repeat MR-HC metabolic assessments (data shown in **Tables 1** and **2** **).**

### Biochemistry

For all fasting serum biochemical analytes, there were no significant differences between baseline (HC) and follow-up (MR-HC) measurements (data shown in **Table 1**).

## Discussion

We report on the most highly metabolically-phenotyped study to date on comparison of the metabolic effects of HC versus MR-HC in adult patients with AI. We demonstrate that compared with HC therapy, switching to MR-HC for a period of 3 months associates with significant metabolic benefits, including reduction of fat mass and body fat percentage, and a significant augmentation in SMR.

Assuming a mean average of 6 h sleep per night for each participant and that the augmenting effect of MR-HC on SMR occurs following its initiation, the cumulative effects of the increased SMR during the 3-month study period would have been approximately 1,800 kcal. If we assume that 3,500 kcal equates to 0.45 kg of fat (22), then the increase in SMR with MR-HC over a 3-month period would equate to approximately 0.25 kg of fat mass reduction. This represents a small proportion (just 8%) of the overall reduction in fat mass demonstrated with MR-HC therapy. Therefore, although increased SMR may have contributed towards reduced fat mass with MR-HC therapy, it seems unlikely that this effect alone would have played a major role in reducing fat mass. Exploration of other mechanisms (including appetite regulation) that underlie reduced fat mass with MR-HC in AI, including measurement of fat depot changes (through, for example, Magnetic Resonance Imaging) should form a focus for future research. The equivalence of SMR per unit of lean mass between HC and MR-HC assessments suggests that the numerical increase (albeit non-significant) in lean mass during the 3-month period on MR-HC may underlie the overall increase in SMR with MR-HC compared to that with HC.

To our knowledge, our study is the first report of a direct comparison between HC and MR-HC therapies in AI using WBC-based indirect calorimetry, and the first to show a significant reduction in fat mass with MR-HC compared with HC. Isidori and colleagues reported on a single-blind randomized controlled trial in AI, with treatment options including continued HC versus switch to MR-HC (16). At 24 weeks, there was a significant reduction in body weight for the MR-HC group (mean treatment difference of 4 kg). There was also an improvement in immune cell profiles, reduced susceptibility to infections and improved quality of life in those randomized to MR-HC compared to HC (16).

Prior studies on the effect of exogenous glucocorticoids on energy expenditure have only focused on supra-physiological replacement doses in patients without AI. In one such study on healthy female volunteers who had 1 mg of betamethasone given orally twice a day for 21 days, there was a significant mean increase in fat mass of 1.5 kg (23). There was also a significant 26% increase in total energy expenditure using a doubly-labeled water method (23). The authors hypothesized that increased dietary energy intake was the most likely explanation for the increase in fat mass (despite increased energy expenditure), in response to supra-physiological steroid doses in healthy volunteers (23). In a further placebo-controlled study on healthy male volunteers, supra-physiological administration of exogenous glucocorticoids (intravenous infusion of methylprednisolone and oral prednisolone) during a weight-maintenance diet resulted in increased energy expenditure (measured through indirect calorimetry) (24). There was also a significant increase in energy intake in response to exogenous glucocorticoid administration. The authors hypothesized a possible effect of glucocorticoids on the central regulation of appetite (24).

Data from these previous studies that explored the metabolic effects of supra-physiological doses of glucocorticoids in healthy volunteers, and data from our own study on the metabolic effects of GRTs in AI, are not directly comparable. However, it is possible that differences in central appetite regulation between HC and MR-HC can be extrapolated to AI, mediated through differences in pharmacokinetic 24-h steroid profiles between these two forms of GRT, and consequent differences in steroid exposure of the central appetite centers. In one study, it was shown that compared with dose-equivalent thrice-daily HC, MR-HC associates with a 20% reduction in the area-under-the-curve for 24-h serum cortisol levels (25). However, this change in overall steroid exposure needs to be countered with a profile of serum cortisol that is more physiologically replicable for MR-HC than for HC. Possible changes in appetite and caloric intake between MR-HC and HC in patients with AI remains entirely speculative, and should form a focus for future research. Furthermore, future comparisons between HC and MR-HC in AI should include groups with equivalent daily exposure to GRT (to take account of the 20% reduction in the daily corticosteroid exposure for MR-HC, compared with the equivalent daily dose of HC (25)). This would require a dose adjustment for MR-HC, to explore further the mechanisms that underlie the metabolic differences between HC and MR-HC in AI.

One potential explanation for the metabolic benefits conferred by MR-HC vs standard HC therapy in our study relates to improved regulation of circadian clock mechanisms. Glucocorticoids mediate interactions between central and peripheral clocks in humans (26). Adrenal disorders such as AI associate with dysregulation of clock synchronization through disrupted circadian rhythms of cortisol (26). Furthermore, evidence suggests that replicating a more physiological cortisol rhythm through once-daily MR-HC rather than multiple daily doses of HC confers additional benefits that include restoration of immune function and improved inflammatory profile (16). These benefits correlate with improved circadian gene expression profile (19). Switching from multiple daily HC administration to once-daily MR-HC associates with improved synchronization of clock gene expression (26). Furthermore, improved glucocorticoid exposure during the night with MR-HC may result in improved synchronization of nocturnal autonomic pathways (27), and optimized entrainment of hypothalamic and melanocortinergic activities (26). We hypothesize that such nocturnal re-synchronization of key neuro-autonomic and endocrine pathways underlies, at least in part, the increase in SMR observed following switch from HC to MR-HC therapy.

Our study has some limitations. Inclusion of a larger number of participants would have provided better power for detection of other possible metabolic differences between HC and MR-HC in AI. However, our usage of the paired t-test with comparisons of baseline HC and repeat MR-HC for each participant would have limited any potentially confounding differences between participants. Furthermore, we did not perform detailed assessments of dietary intake, appetite, and physical activity that may have provided more insights into the improved fat mass with MR-HC. Furthermore, our study was of a relatively short duration, and future studies should focus on the possible longer-term metabolic effects (beyond 3 months) of MR-HC in AI, and the durability of any early metabolic changes such as reduced fat mass and augmented SMR. It was not possible to perform sub-group analyses on our data due to a relative lack of power. With inclusion of larger numbers of participants, with a greater range of BMI, future studies should explore any predictors (such as baseline BMI or fat mass) for the metabolic benefits of MR-HC in patients with AI. Due to the pilot and observational nature of our study (with a single crossover design), our study lacked any placebo arm or randomization, with the possibility (although unlikely) of an order effect. Finally, as the HC and MR-HC metabolic assessments occurred 3 months apart, seasonal differences may have potentially confounded our metabolic data.

To summarize, we demonstrate that in adult patients with AI who take multiple daily doses of HC therapy, switching to MR-HC results in a significant reduction in body fat and augmentation of SMR within 3 months. It is possible that increased SMR in response to MR-HC may contribute towards a favorable effect on fat mass, although this mechanism is unlikely to play a major role. The factors that underlie the metabolic benefits of MR-HC compared with HC in patients with AI should form a focus for future studies in larger numbers of participants, with more detailed phenotyping (including data on dietary intake, physical activity, fluid status, blood pressure, muscle mass, fat distribution, and glycemic control) and for a longer duration. Given the importance of obesity and metabolic dysfunction as a modern-day global health issue that underlies much 21st century chronic ill-health (including in patients with AI), any improvement in fat mass and metabolic status is likely to confer substantial clinical benefits on a population level that would likely improve overall metabolic health and health economic efficiency. Our data support the use of MR-HC in adult patients with AI. Such an approach for the management of AI should be considered in future guidelines.

## Data Availability Statement

The original contributions presented in the study are included in the article/supplementary material. Further inquiries can be directed to the corresponding author.

## Ethics Statement

The studies involving human participants were reviewed and approved by West Midlands Black Country Research Ethics Committee, UK. The patients/participants provided their written informed consent to participate in this study.

## Author Contributions

This research study protocol was designed by TB, MW, and AG. Execution of the study was led by CB and DB, with input from PH. Data analysis was conducted by CB, JH, and TB. All authors contributed to the preparation of this manuscript, and write-up was mainly coordinated by TB, with input from CB, AG, MW, and HR. All authors contributed to the article and approved the submitted version.

## Funding

This Investigator-Initiated Research was supported by a grant from Shire International GmbH, a Takeda company (grant number IST-GBR-000735), and an NIHR Academic Clinical Fellowship.

## Conflict of Interest

The authors declare that the research was conducted in the absence of any commercial or financial relationships that could be construed as a potential conflict of interest.

This publication presents independent research carried out with the support of the National Institute of Health Research (NIHR) Coventry and Warwickshire Clinical Research Facility. The views expressed are those of the author(s) and not necessarily the NHS, the NIHR or the Department of Health.

The authors declare that this study received funding from Shire International GmbH. The funder had no role in the study design, data collection and analysis, decision to publish or preparation of the manuscript.

## Abbreviations

AI, adrenal insufficiency; BMI, body mass index; BW, body weight; DM, diabetes mellitus; GRT, glucocorticoid replacement therapy; HC, hydrocortisone; HMRU, Human Metabolism Research Unit; HPA, hypothalamo-pituitary adrenal; IQR, Inter-quartile range; LM, lean mass; MR-HC, modified-release hydrocortisone; NS, non-significant; SD, standard deviation; SMR, sleeping metabolic rate; T1D, type 1 diabetes mellitus; T2D, type 2 diabetes mellitus; TMR, total metabolic rate; UHCW, University Hospitals Coventry and Warwickshire; WBC, whole body calorimeter; WISDEM, Warwickshire Institute for the Study of Diabetes, Endocrinology and Metabolism.
